# Supplementary material for: Aspect Ratio Controls Hot-Carrier Generation in Gold Nanobricks
Source: J Phys Chem C Nanomater Interfaces. 2025 Feb 27;129(10):4886–92. doi: 10.1021/acs.jpcc.4c08595 (PMC11912479; doi:10.1021/acs.jpcc.4c08595)
Supplement: Supplementary file 1 — jp4c08595_si_001.pdf [file jp4c08595_si_001.pdf]

Supplementary Information for Aspect Ratio Controls Hot Carrier Generation in Gold Nanobricks

Simão M. João,[†] Ottavio Bassano,[†] and Johannes Lischner[*,†,‡]

†*Department of Materials, Imperial College London, South Kensington Campus, London SW7 2AZ, United Kingdom*

‡*The Thomas Young Centre for Theory and Simulation of Materials, London E1 4NS, United Kingdom*

E-mail: j.lischner@imperial.ac.uk

1 Polarization average

Under the quasi-static approximation, the solution to Laplace's equation for a general external field $\mathbf{E}$ can be constructed from the solution to fields along the three Cartesian axes. Let Φ_x, Φ_y, Φ_z be the electric potential resulting from an electric field $\mathbf{E}_x, \mathbf{E}_y, \mathbf{E}_z$ along the x, y, z directions respectively. Then, the electric potential induced by a generic field $\mathbf{E} = \alpha\mathbf{E}_x + \beta\mathbf{E}_y + \gamma\mathbf{E}_z$ is simply $\Phi = \alpha\Phi_x + \beta\Phi_y + \gamma\Phi_z$. Setting α, β, γ to be the direction cosines and $\mathbf{E}_x, \mathbf{E}_y, \mathbf{E}_z$ to be unit vectors, the potential is written as

$$\Phi\left(\theta, \phi\right) = \sin\theta\cos\phi\Phi_x + \sin\theta\sin\phi\Phi_y + \cos\theta\Phi_z$$

and the polarization-averaged electron generation rate requires an average over the polar

angle

$$N_e\left(E,\omega\right)\sim\int_0^{\pi}d\theta\int_0^{2\pi}d\phi\sin\theta\mathrm{Tr}\left[\delta\left(\varepsilon-H_0\right)\Phi\left(\theta,\phi\right)\delta\left(E-H_0\right)\Phi^{\dagger}\left(\theta,\phi\right)\right]$$

The angular integrals can be solved analytically, showing that the average over polarization is identical to averaging over the three cartesian directions:

$$N_e\left(E,\omega\right)\sim\sum_{i=x,y,z}\mathrm{Tr}\left[\Phi_i^{\dagger}\delta\left(\varepsilon-H_0\right)\Phi_i\delta\left(E-H_0\right)\right]$$

Furthermore, having defined z as the long axis and x, y as the short axes of the nanobricks, only the solutions along x and z are required since the nanobricks are symmetrical with respect to 90º rotations around the z axis.

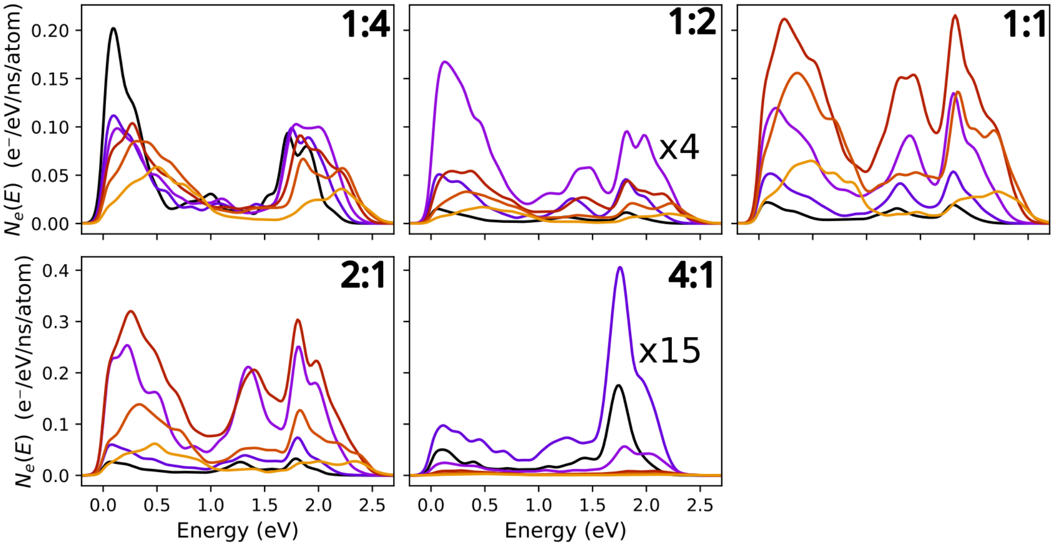

Figure S1: Electron generation rate for nanobricks with aspect ratios ranging from 1:4 to 4:1, averaged over all light polarizations. Each curve represents a different light frequency, from 2.0 eV to 2.5 eV.

Figure S1 represents the rate and energy at which electrons are being generated for different illumination frequencies and aspect ratios. This Figure is the polarization-averaged (or, equivalently, orientation-averaged) analogue of Fig. 2 in the main text and would be represen-

tative of the hot carrier generation rate of a distribution of randomly-oriented nanoparticles, such as in solution. Comparing to Fig. 2, the polarization averaged HCG for aspect ratios between 1:4 and 1:2 is dominated by interband transitions arising from the long nanoparticles with their axis aligned with the electric field. In contrast, the 2:1 and 4:1 aspect ratios are dominated by intraband transitions coming from flat nanoparticles whose short axis (x) is parallel to the electric field.

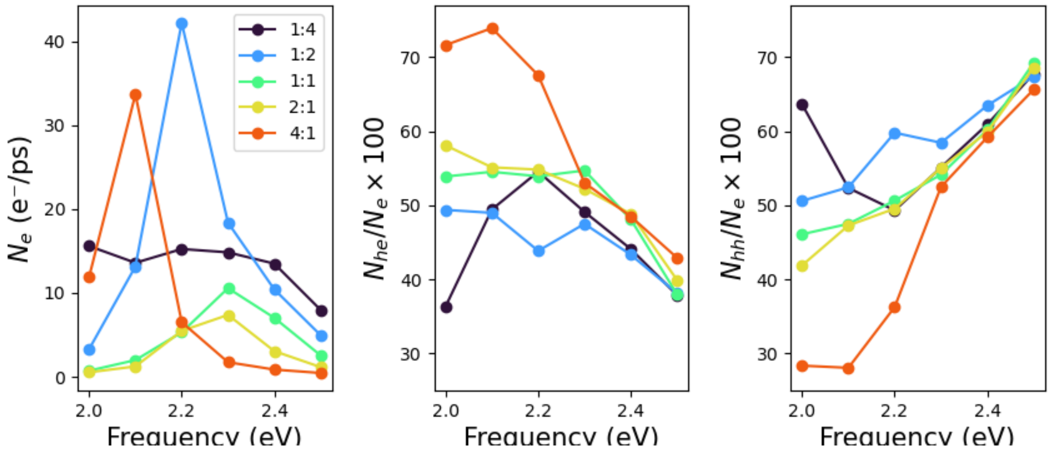

Figure S2: Total electron generation rate (left) and percentage of hot electrons (middle) and hot holes (right), as a function of illumination frequency averaged over all light polarizations. The curves are color-coded according to the main text.

Figure S2 shows the total rate of electron generation N_e, as well as the ratio of hot electrons and holes to total electrons. N_e attains the largest values for frequencies of 2.1 and 2.2 eV for the aspect ratios 4:1 and 1:2 respectively. As a general trend, the fraction of hot holes (electrons) tends to decrease (increase) as the frequency increases. The 4:1 AR shows the highest overall sensitivity in the proportion of hot electrons/holes to changes in frequency.
